# Mitigating cadmium and salinity stress in lettuce through biochar application: impacts on growth and nutrient dynamics

**DOI:** 10.1186/s12870-025-08051-y

**Published:** 2026-01-07

**Authors:** Hüseyin Eren Korkmaz, Mehmet Akgün, Ayhan Kocaman, Kürşat Korkmaz

**Affiliations:** 1 Cerrahpaşa Faculty of Medicine, Istanbul University, Istanbul, TR34098 Turkey; 2 Giresun University, Rectorate, Hazelnut Specialization Coordinatorship, Giresun, TR28200 Turkey; 3 Environmental Engineering Department, Karabük University Engineering Faculty, Karabük, TR78050 Turkey; 4 Department of Soil Science and Plant Nutrition, Faculty of Agriculture, Ordu University, Ordu, TR52100 Turkey

**Keywords:** Salinity, Immobilization, Heavy metal, *Lactuca sativa* L.

## Abstract

Heavy metal contamination and soil salinity pose significant threats to safe and sustainable vegetable production, particularly for sensitive crops such as lettuce. These combined stresses hinder nutrient uptake, accelerate toxicity, and underscore the need for effective soil amendments that can mitigate multi-stress conditions. However, the potential of low-cost, hazelnut husk–derived biochar to alleviate simultaneous cadmium (Cd) and salinity stress in lettuce remains poorly understood. This study evaluated the effects of biochar applications on lettuce (*Lactuca sativa* L.) grown under varying cadmium (Cd) concentrations and salt stress conditions. A greenhouse experiment was conducted using a factorial design with four levels of biochar (0, 0.5, 1, and 2%), four Cd treatments (0, 0.5, 1, and 2 mg kg⁻¹), and two salinity regimes (0 and 75 mM NaCl), with three replicates per treatment. Plant growth, yield, and the concentrations of macro-nutrients phosphorus (P), potassium (K), calcium (Ca), magnesium (Mg), and sodium (Na), and micro-nutrients iron (Fe), copper (Cu), manganese (Mn), and boron (B), as well as Cd, were determined. Results showed that biochar significantly increased shoot biomass, improved nutrient concentrations, and reduced Cd accumulation in lettuce shoots. Under non-saline conditions, the highest biochar dose (2%) reduced shoot Cd concentrations by approximately 30–50% across Cd levels and increased shoot biomass by about 40–50% compared with the non-amended control. Under saline conditions, biochar still decreased shoot Cd concentrations by around 25–30% and increased shoot dry weight by up to nearly 60%. Across Cd and salinity levels, biochar markedly reduced shoot Na concentrations (by almost 90%) and enhanced K⁺, Mn, and B concentrations; under salt stress, Zn increased by approximately 40%. Principal Component Analysis (PCA) revealed that PC1 primarily reflected gradients associated with the effects of biochar and Cd on nutrient and Cd profiles, whereas PC2 captured variability driven by salinity. Overall, these findings demonstrate that hazelnut husk–derived biochar is an effective and low-cost soil amendment that can improve nutrient status, reduce Cd bioavailability, and mitigate salinity-induced ionic imbalances, offering a practical strategy for enhancing lettuce production in Cd-contaminated and saline soils.

**Graphical Abstract:**

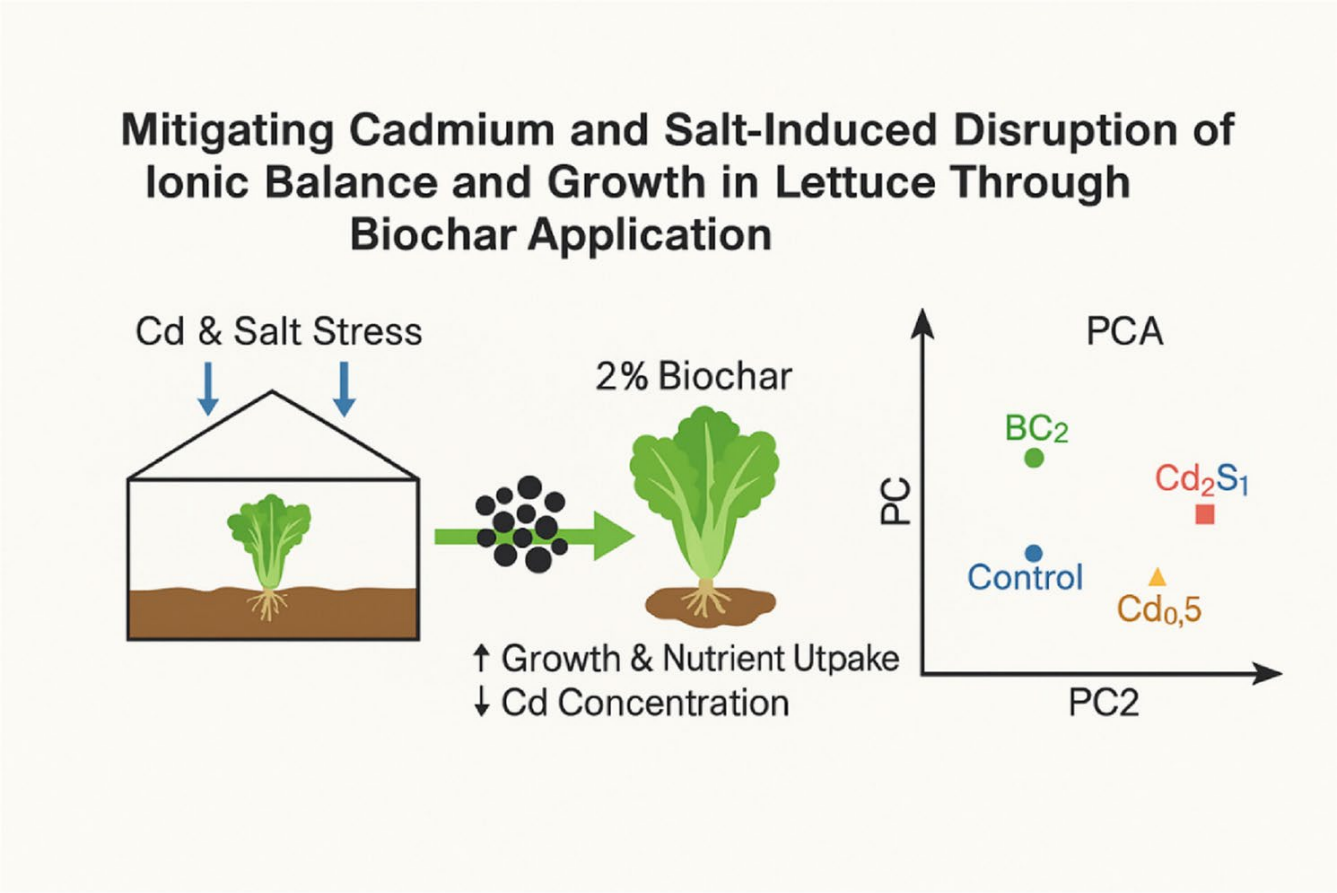

## Introduction

Environmental stresses on agricultural soils have been exacerbated by industrialization, population growth, and unsustainable agricultural practices, which have led to a significant deterioration in soil quality and productivity [1, 2]. Among these stresses, soil salinity and heavy metal contamination have become particularly urgent challenges that necessitate integrated and sustainable soil management strategies. Anthropogenic influences, especially the excessive use of synthetic fertilizers, are increasingly causing chemical imbalances in the soil and contributing to salinity, which in turn exacerbates the degradation processes in agricultural soils [3–8]. The excessive use of chemical fertilizers is one of the main causes of soil salinization and the accumulation of heavy metals [8–12]. In areas with intensive irrigation, salinization problems are exacerbated and affect soil health and crop productivity [13, 14]. The continuous use of chemical fertilizers disturbs the nutrient balance in the soil and increases salinity, which in turn affects plant growth and yield [4, 15–17]. Research has shown that the excessive use of fertilizers contributes to salinization and the deterioration of the physical and chemical properties of the soil [18, 19]. High concentrations of soluble salts cause osmotic stress in plants, which limits nutrient uptake and reduces productivity [14]. This can trigger a feedback loop in which declining fertility increases dependence on fertilizers and exacerbates salinity [20]. Contamination with heavy metals, especially cadmium, also poses a considerable risk [21]. Cadmium and other metals such as lead and arsenic can accumulate in the soil through fertilizers or additives, and these metals not only affect soil health but also pose a risk to food safety if they enter the food chain [22, 23]. Salinity can increase the mobility and bioavailability of heavy metals, which promotes their uptake by plants [24, 25] and makes remediation measures more difficult [19, 26]. The interaction between salinity and heavy metal pollution makes agricultural management even more difficult.

Heavy metal contamination and soil salinity are among the most critical environmental constraints limiting agricultural productivity and food quality, particularly in leafy vegetables such as lettuce (*Lactuca sativa L*.). Lettuce is highly sensitive to ionic imbalance, osmotic stress, and metal-induced toxicity, leading to reduced biomass, impaired nutrient uptake, and poor physiological performance [27, 28]. These stressors reduce yield, impair quality, and increase physiological damage [29–32]. Additionally, cadmium and salinity interact synergistically, intensifying toxicity and nutrient imbalance [33]. Biochar has proven to be a promising soil conditioner that can mitigate both salinity and heavy metal contamination [34]. Produced by the pyrolysis of organic materials, biochar improves performance in contaminated soils. Recent comparative studies suggest that cadmium and salinity soil structure enhance nutrient retention and immobilize heavy metals [35, 36]. Its porous structure increases water-holding capacity and supports salt leaching, while biochar-induced pH changes may improve nutrient availability only within an optimal pH range, as excessive alkalinity can decrease the solubility of micronutrients such as Fe, Zn, Mn, and Cu [37–39]. Studies have shown that biochar reduces the bioavailability of cadmium primarily through cation adsorption mechanisms, which depend on pyrolysis conditions and feedstock characteristics [36, 40–42]. In this context, the amorphous and porous carbon matrix with a large surface area facilitates the adsorption of cations such as Na⁺ and Cd²⁺, thereby reducing their mobility and uptake by plants [36, 43]. In addition to biochar, various organic amendments such as compost, manure, vermicompost, and materials derived from press mud or straw have been utilized to immobilize heavy metals and enhance plant stress in lettuce, focusing on growth performance, nutrient dynamics, and Cd accumulation. Additionally, hazelnut husk was selected as the biochar feedstock due to its abundance as an agricultural by-product in the Black Sea region, low cost, and previous evidence supporting its favorable physicochemical properties (e.g., high surface area and nutrient retention capacity). While these organic materials can mitigate metal phytotoxicity and promote growth, biochar and biochar–compost mixtures often result in more significant and sustained reductions in extractable Cd and leaf metal concentrations, particularly in leafy vegetables and cereals [44]. Given the combined physiological constraints imposed by Cd toxicity and salinity, there is a need for effective soil amendments that can mitigate both stresses simultaneously. Despite significant advancements, several knowledge gaps persist. Most studies have concentrated on either Cd toxicity in isolation or salinity as a singular stress factor. Very few investigations have explored the combined effects of these factors on lettuce, which is particularly susceptible to ionic and oxidative stress. Additionally, the role of hazelnut husk–derived biochar in simultaneously modulating Cd uptake, Na accumulation, and a wide range of macro- and micronutrients under dual stress conditions remains inadequately understood. Furthermore, there is limited information regarding how different doses of this specific biochar affect nutrient stoichiometry and plant ion homeostasis across varying salinity levels. Consequently, the present study aimed to: (i) assess the effectiveness of hazelnut husk–derived biochar in alleviating the detrimental effects of combined Cd and salinity stress on lettuce growth, nutrient dynamics, and Cd accumulation under both non-saline and saline conditions; and (ii) clarify how increasing applications of biochar influence nutrient interactions and ionic balance, aided by Principal Component Analysis (PCA). We hypothesized that increasing the dose of hazelnut husk biochar would reduce Cd uptake and Na accumulation and improve nutrient balance and growth under both saline and non-saline conditions, with the strongest effects at higher application rates.

## Materials and methods

### Soil and Biochar properties

Soil samples were collected from a depth of 20 cm within the research farm, followed by air drying and sieving through a 2 mm mesh to prepare the soil for analysis. In the soil samples, texture was determined using the Bouyoucos hydrometer method [45], soil pH was measured using a 1:2.5 dilution method [46], electrical conductivity (EC) was assessed with a 1:2.5 dilution method [47], organic matter content was determined following the method of Walkley and Black [48]. Available phosphorus was determined using the method of Bray and Kurtz [49]. Available phosphorus was determined according to Pratt [50]. Analyses of Fe, Cu, Zn, Mn, and Cd were performed using the DTPA extraction method developed by Kacar [51], and the results are presented in Table 1.

Biochar material was obtained by heating hazelnut husk by “slow pyrolysis” (at < 400 °C) without oxygen. Hazelnut husk was dried in an oven at 60 °C for 1 day with less than 10% moisture concentration and transferred to the biochar oven. In biochar production, the oven temperature increased by 10 °C min^-1^ and was kept at 400 °C for 2 h and made ready for application [52]. Following the cooling process, the biochar obtained was subjected to grinding and subsequently sieved to achieve a particle size of less than 2 mm. The pH and electrical conductivity (EC) of biochar were determined in 1:5 biochar to deionize water suspension and extract, respectively. Total concentrations of P, K, Zn, Mn, Fe, Cu, and Cd in biochar were determined after dry ashing 0.5 g samples at 550 °C for 6 h, followed by acid digestion of the ash, and analyzed using atomic absorption spectrometry (AAS) [53, 54]. While the hazelnut husk biochar contained 0.41 mg of Cd per kg (as shown in Table 1), its effect on the overall Cd concentration in the soil pots was minimal when compared to the experimental Cd additions. Even at the maximum application rate of 2% biochar, the Cd contribution from the biochar was approximately 0.008 mg Cd per kg of soil, accounting for less than 2% of the lowest Cd treatment (0.5 mg kg⁻¹). Therefore, the inherent Cd content of the biochar was not anticipated to significantly impact the assessment of Cd immobilization efficiency; nonetheless, this background contribution was considered when interpreting the results.

**Table 1:** Some physical and chemical properties of Biochar and soil used in the experiment

	Soil	Biochar		Soil	Biochar
Texture	Sandy Loam (SL)		P (mg kg^− 1^)	8.12	154.1
Sand (%)	43.7		K (mg kg^− 1^)	112.1	8302.2
Silt (%)	30		Fe (mg kg^− 1^)	5.42	277
Clay (%)	25.3		Cu (mg kg^− 1^)	2.4	14.2
pH (1/2.5)	6.68	7.64	Zn (mg kg^− 1^)	0.4	45.6
EC (µS m^− 1^)	274.2	175.4	Mn (mg kg^− 1^)	22	56.2
Organic Matter (%)	0.68	98.1	Cd (mg kg^− 1^)	0.002	0.41

### Greenhouse experiment

A greenhouse experiment was conducted at the Ordu University Faculty of Agriculture Research Farm in 2022 to investigate the effects of cadmium and biochar amendments derived from hazelnut husks on lettuce (*Lactuca sativa L*., cv. *Fanela*) cultivation under saline conditions. Lettuce seeds were obtained from AG Tohum (Antalya, Turkey), a certified commercial seed company. The greenhouse experiment was conducted using non-sterilized soil containing native microbiota. Each pot was filled with 3 kg of soil, and one lettuce seedling was transplanted per pot. The experiment was carried out in a controlled greenhouse environment characterized by relative humidity levels ranging from 60% to 80%, natural day/night light conditions, and average temperatures of 20 °C during the day and 15 °C at night. The experimental design used a factorial arrangement with four different cadmium doses (0, 0.5, 1, and 2 mg Cd kg^− 1^) applied as cadmium nitrate (Cd (NO₃)₂), four biochar concentrations (0, 0.5, 1, and 2%), and two levels of salt stress (0 and 75 mM NaCl), each replicated three times. Cadmium doses were designated as Cd0, Cd0.5, Cd1, and Cd2, representing 0, 0.5, 1, and 2 mg Cd kg^− 1^, respectively. Biochar treatments were labeled BC0, BC0.5, BC1, and BC2, corresponding to 0%, 0.5%, 1%, and 2% biochar. Salt stress levels were coded as S0 (0 mM NaCl) and S1 (75 mM NaCl).

Biochar was applied to the soil 30 days prior to transplanting and incubated during this period to stabilize the soil. Cd was added at the beginning of the incubation period, allowing it to interact with both soil and biochar throughout the 30-day incubation. During this time, soil moisture was maintained at 60–70% of field capacity by adding distilled water and mixing weekly at ambient temperature (23 ± 2 °C), ensuring equilibration and proper distribution of Cd in the soil-biochar matrix before transplanting. Salt stress was initiated at the time of transplanting and gradually increased by weekly additions of 25 mM NaCl to reach the final concentration of 75 mM at the end of the third week. All treatments were thus applied during the early vegetative stage of lettuce development, allowing stress responses to be observed from the onset of growth.

Basic fertilizers were applied post-planting to ensure optimal nutrient availability for the seedlings. Specifically, nitrogen was supplied at a rate of 200 mg N kg^− 1^ in the form of ammonium nitrate (NH₄NO₃), phosphorus at 100 mg P kg^− 1^ as potassium dihydrogen phosphate (KH₂PO₄), potassium at 125 mg K kg^− 1^ also from KH₂PO₄, and zinc at 2.5 mg Zn kg^− 1^ sourced from zinc sulfate heptahydrate (ZnSO₄·7 H₂O). Each pot contained one lettuce seedling to facilitate accurate assessment of growth responses under varying treatment conditions.

During the experimental period, pots were weighed every other day under greenhouse conditions to maintain field capacity, and any water loss was compensated for by irrigation with distilled water. The plant growth period was determined based on the severity of chlorosis and growth differences observed in the control groups under salt stress, leading to harvest at the end of two months. The harvested plants were first washed with tap water and subsequently rinsed with distilled water. They were then dried in an oven at a constant temperature of 70 °C for 48 h until reaching a constant weight, allowing for the determination of dry biomass. Following drying, the plant samples were ground to prepare them for further analysis. Plant samples were digested using a microwave-assisted acid digestion procedure using a mixture of HNO₃ and H₂O₂ (5 mL HNO₃ and 2 mL H₂O₂ at a 5:2 v/v ratio). Each digestion batch included reagent blanks, and certified reference material (CRM), specifically NIST SRM 1573a or a comparable plant matrix CRM, was used to verify analytical accuracy. Recoveries for all measured elements fell within acceptable limits (90–110%), and the concentrations of Cd, P, K, S, Ca, Mg, Na, Zn, Fe, Cu, Mn, and B in the digested samples were determined using ICP-OES (Perkin Elmer, 2100 DV Optima) [55].

### Statistical analysis

Data were analyzed using a three-way ANOVA in SPSS software (version 22.0). When ANOVA revealed significant main or interaction effects, Duncan’s Multiple Range Test was employed for mean separation and letter-based grouping at the *p* < 0.05 level. Additionally, the Least Significant Difference (LSD) test was utilized to further assess treatment differences at stricter significance thresholds (*p* < 0.01 and *p* < 0.001). Means that are accompanied by different letters indicate statistically significant differences as determined by Duncan’s test (*p* < 0.05), while the LSD test was applied to confirm high-level significance for selected comparisons. Values that lack letters suggest no significant difference (*p* > 0.05). Furthermore, Principal Component Analysis (PCA) was conducted to evaluate the relationships among nutrient dynamics under the combined stress of salinity and cadmium, together with biochar applications.

## Results

### Effects of biochar, cadmium, and salinity on shoot biomass and Cd accumulation

The interactive effects of biochar (BC), Cd, and salinity (NaCl) on lettuce fresh and dry shoot biomass are presented in Table 2. Both fresh and dry biomass were significantly influenced by the combined effects of BC, Cd, and salt stress (*p* < 0.05), highlighting the importance of interaction effects on plant growth.

Under non-saline conditions (0 mM NaCl), fresh shoot weight increased consistently with BC dose across all Cd levels. In the absence of biochar (BC0), increasing Cd dose did not lead to a reduction in biomass; instead, fresh shoot weight rose from 46.70 g plant⁻¹ at Cd0 to 99.13 g plant⁻¹ at Cd2, indicating that growth was maintained or even slightly stimulated within the Cd range tested (Table 2). The lowest biomass was observed in the control without biochar and Cd (BC0–Cd0: 46.70 g plant^− 1^), whereas the highest fresh shoot weight was recorded at BC2–Cd2 (124.20 g plant^− 1^), corresponding to a 166% increase relative to the control. Intermediate BC doses (0.5% and 1%) also enhanced fresh shoot weight, with BC1–Cd2 reaching 108.87 g plant^− 1^ (≈ 133% increase) and BC0.5–Cd2 reaching 102.60 g plant^− 1^ (≈ 120% increase). Dry shoot weight showed similar trends: the control (BC0–Cd0) exhibited 2.72 g plant^− 1^, while BC2–Cd2 achieved 6.33 g plant^− 1^, a 133% increase. The positive effect of higher BC doses on biomass was particularly evident at elevated Cd levels, suggesting that biochar application improved plant growth under Cd exposure. The increase in biomass with higher BC doses was more pronounced at elevated Cd levels, indicating that biochar mitigates Cd-induced growth inhibition, likely by immobilizing Cd and improving nutrient availability.

Under saline conditions (75 mM NaCl), fresh shoot weight in the control (BC0–Cd0) was severely reduced to 10.30 g plant^− 1^, representing an 78% reduction relative to the non-saline control. Within the BC0 treatments under salinity, fresh biomass remained low but did not markedly decline with increasing Cd dose, varying only slightly between 10.30 and 11.13 g plant⁻¹ across Cd0–Cd2 (Table 2). However, BC application alleviated salt stress in a dose-dependent manner. The highest fresh shoot weight under salinity was observed in BC2–Cd0 (17.53 g plant^− 1^), corresponding to a 70% increase over the BC0 control. For Cd-exposed plants under salinity, BC2 increased fresh shoot weight from 11.13 g plant^− 1^ (BC0–Cd2) to 15.90 g plant^− 1^ (BC2–Cd2), a 43% increase, demonstrating that biochar partially offsets the combined inhibitory effects of Cd and salt. Dry shoot weight under saline conditions followed a similar pattern: BC2–Cd0 reached 1.63 g plant^− 1^ compared to 1.01 g plant^− 1^ in the control (≈ 61% increase), whereas BC2–Cd2 improved dry weight to 1.45 g plant^− 1^ from 1.13 g plant^− 1^ in BC0–Cd2 (≈ 28% increase). Intermediate BC doses (0.5% and 1%) also enhanced both fresh and dry biomass, particularly at lower Cd levels, further indicating a dose-dependent mitigation of salinity and Cd stress. Overall, biochar application markedly improved both fresh and dry shoot biomass under non-saline and saline conditions, with the strongest effects observed at the highest dose (BC2). The interactive effects of biochar, Cd, and salinity indicate that biochar not only promotes growth under non-saline conditions but also helps to maintain biomass and partially mitigate growth constraints under combined Cd and salt stress. The interactive effects of biochar, Cd, and salinity indicate that biochar not only promotes growth under normal conditions but also mitigates Cd- and salt-induced growth inhibition. The relative improvements were most pronounced under non-saline conditions and at higher Cd levels, whereas under salinity, biochar provided partial mitigation, emphasizing its potential as a sustainable soil amendment to enhance lettuce productivity under multiple environmental stresses.

Analysis of variance revealed statistically significant effects (*p* < 0.05) of Cd concentration, BC dose, and salt stress on Cd accumulation in lettuce shoots, as well as significant interactions among these factors (Table 3). Cd concentrations in the shoots increased consistently with increasing soil Cd levels under both non-saline (S0) and saline (S1) conditions. In the non-saline control (BC0), shoot Cd ranged from 0.47 mg kg^− 1^ at Cd0 to 5.69 mg kg^− 1^ at Cd2, demonstrating a clear dose-dependent accumulation pattern.

Biochar amendment significantly mitigated Cd uptake, particularly at the highest dose (BC2). Under non-saline conditions, BC2 reduced shoot Cd by approximately 30% at Cd0.5 (from 2.94 to 2.05 mg kg^− 1^), 48% at Cd1 (from 4.62 to 2.39 mg kg^− 1^), and 43% at Cd2 (from 5.69 to 3.26 mg kg^− 1^) compared to unamended controls. Intermediate biochar doses (BC0.5 and BC1) also decreased Cd levels, though less markedly, indicating a dose-dependent immobilization effect.

**Table 2:** Effects of Biochar applications on fresh and dry shoot weight of lettuce plants under salt and cd applications

NaCl doses (mM)	Biochar doses (%)	Cd levels (mg kg^− 1^)			
		0	0.5	1	2
Fresh Shoot Weight (g plant^− 1^)					
0	**0**	46.70 ± 1.30	72.73 ± 2.91	87.70 ± 23.32	99.13 ± 12.80^b^
	**0.5**	66.73 ± 13.39^a*^	76.03 ± 3.20	88.40 ± 9.63	102.60 ± 17.51^ab^
	**1**	73.53 ± 0.42^a**^	84.90 ± 19.10	93.80 ± 8.20	108.87 ± 1.25^ab*^
	**2**	76.47 ± 9.16^a**^	87.20 ± 12.60	107.80 ± 3.20	124.20 ± 4.20^a***^
75	**0**	10.30 ± 1.50^c^	11.27 ± 1.68^b^	12.20 ± 0.80	11.13 ± 1.33^b^
	**0.5**	14.40 ± 1.80^b**^	15.80 ± 3.20^a*^	12.30 ± 0.10	11.50 ± 1.90^b^
	**1**	15.80 ± 1.00^ab***^	17.07 ± 1.42^a*^	12.70 ± 1.90	13.40 ± 0.20^b**^
	**2**	17.53 ± 1.33^a***^	16.30 ± 2.30^a*^	12.07 ± 0.99	15.90 ± 0.10^a***^
Dry Shoot Weight (g plant ^− 1^)					
0	**0**	2.72 ± 0.29^b^	3.59 ± 0.17	4.04 ± 0.84	4.24 ± 0.31^b^
	**0.5**	3.90 ± 0.80^a*^	3.90 ± 1.09	4.27 ± 0.02	4.43 ± 0.59^b^
	**1**	4.13 ± 0.32^a**^	4.10 ± 0.46	4.31 ± 0.04	4.52 ± 1.01^b^
	**2**	3.95 ± 0.36^a*^	4.87 ± 0.98	4.71 ± 0.40	6.33 ± 0.80^a**^
75	**0**	1.01 ± 0.09^c^	1.09 ± 0.04^c^	1.11 ± 0.18	1.13 ± 0.03^c^
	**0.5**	1.31 ± 0.20^b**^	1.61 ± 0.10^b***^	1.15 ± 0.02	1.21 ± 0.01^bc^
	**1**	1.44 ± 0.01^ab**^	1.78 ± 0.17^ab***^	1.21 ± 0.30	1.27 ± 0.09^b*^
	**2**	1.63 ± 0.21^a***^	1.82 ± 0.07^a***^	1.26 ± 0.07	1.45 ± 0.08^a***^

Means followed by different letters indicate significant differences according to Duncan’s Multiple Range Test (*p* < 0.05). LSD test results at *p* < 0.01 and *p* < 0.001 are also provided for additional pairwise comparisons

^*^: *p* < 0.05; ^**^: *p* < 0.01; ^***^: *p* < 0.001; ns: *p* > 0.05

Under saline conditions (S1), Cd accumulation at low Cd doses (Cd0 and Cd0.5) remained low, while higher Cd levels (Cd1 and Cd2) led to increased shoot Cd compared to corresponding non-saline treatments. For instance, at Cd1, BC1-treated plants accumulated 1.95 mg kg^− 1^ Cd, indicating that Na⁺ ions partially interfered with biochar-mediated Cd immobilization. Nevertheless, BC2 consistently reduced Cd uptake under salt stress, lowering Cd from 2.74 to 2.04 mg kg^− 1^ at Cd2 (~ 26% reduction). These results demonstrate statistically significant main and interaction effects of Cd, biochar, and salinity on Cd accumulation in lettuce. The findings underscore the efficacy of biochar, especially at 2%, in reducing Cd translocation to shoots, although salt stress can modulate this mitigation.

### Effects on micronutrient dynamics (B, Cu, Fe, Mn, Zn)

The interactive effects of BC, Cd, and salinity on lettuce fresh and dry shoot biomass are presented in Table 2. Three-way ANOVA revealed that shoot biomass was significantly affected by the main factors and their interactions (*p* < 0.05), indicating that plant growth responded to the combined influence of BC, Cd, and salinity rather than to single factors alone. Under non-saline conditions (S0), boron (B) concentrations increased with both Cd levels and biochar applications. The lowest B content was recorded in the Cd0 treatment without biochar, measuring 20.95 mg kg^− 1^. Increasing biochar to 2% raised B concentration to 22.70 mg kg^− 1^, an increase of approximately 8%, although this change was not statistically significant. At higher Cd levels, biochar significantly enhanced B accumulation. In the Cd0.5 treatment, B increased from 22.70 mg kg^− 1^ without biochar to 25.91 mg kg^− 1^ with 2% biochar, representing a 14% increase. Similarly, in Cd1, B rose from 23.91 mg kg^− 1^ to 26.29 mg kg^− 1^ with the highest biochar dose, and in Cd2, B increased from 24.87 mg kg^− 1^ to 25.92 mg kg^− 1^. These findings indicate a synergistic effect of biochar and Cd stress in promoting boron uptake under non-saline conditions.

**Table 3:** Effects of Biochar applications on cd concentration in lettuce plants under salt and Cd applications

NaCl doses (mM)	Biochar doses (%)	Cd levels (mg kg^− 1^)			
		0	0.5	1	2
		mg kg^− 1^			
0	**0**	0.47 ± 0.99^b^	2.94 ± 0.46^a**^	4.62 ± 1.47^a*^	5.69 ± 0.73^a*^
	**0.5**	0.38 ± 0.01^b^	2.98 ± 0.53^a**^	4.58 ± 0.88^a*^	5.02 ± 0.15^a*^
	**1**	0.70 ± 0.09^a**^	2.28 ± 0.49^ab^	3.86 ± 0.04^ab^	5.04 ± 1.61^a*^
	**2**	0.68 ± 0.05^a**^	2.05 ± 0.29^b^	2.39 ± 0.37^b^	3.26 ± 0.38^b^
75	**0**	0.37 ± 0.08	0.79 ± 0.26	1.20 ± 0.12^b^	1.69 ± 0.37
	**0.5**	0.41 ± 0.08	0.82 ± 0.10	1.51 ± 0.45^ab^	1.67 ± 0.57
	**1**	0.31 ± 0.08	0.69 ± 0.15	1.95 ± 0.03^a*^	2.74 ± 0.69
	**2**	0.36 ± 0.03	0.51 ± 0.10	1.36 ± 0.10b	2.04 ± 0.67

Means followed by different letters indicate significant differences according to Duncan’s Multiple Range Test (*p* < 0.05). LSD test results at *p* < 0.01 and *p* < 0.001 are also provided for additional pairwise comparisons

^*^: *p* < 0.05; ^**^: *p* < 0.01; ^***^: *p* < 0.001; ns: *p* > 0.05

Under S1 conditions, boron concentrations were considerably lower than in non-saline treatments, reflecting the suppressive effect of salt stress. For instance, in the Cd0 × BC0 group, B dropped from 20.95 mg kg^− 1^ in S0 to 12.72 mg kg^− 1^ in S1, a reduction of approximately 39%. Despite this decline, biochar mitigated boron loss. In the same Cd0 group, application of 2% biochar increased B to 15.53 mg kg^− 1^, corresponding to a 22% improvement over the non-biochar treatment. In the Cd0.5 group, B content increased from 12.24 mg kg^− 1^ without biochar to 14.85 mg kg^− 1^ with 0.5% biochar, while in Cd1 and Cd2, biochar also enhanced B accumulation, reaching the highest value of 13.99 mg kg^− 1^ in BC0.5 × Cd2.

**Fig. 1:**
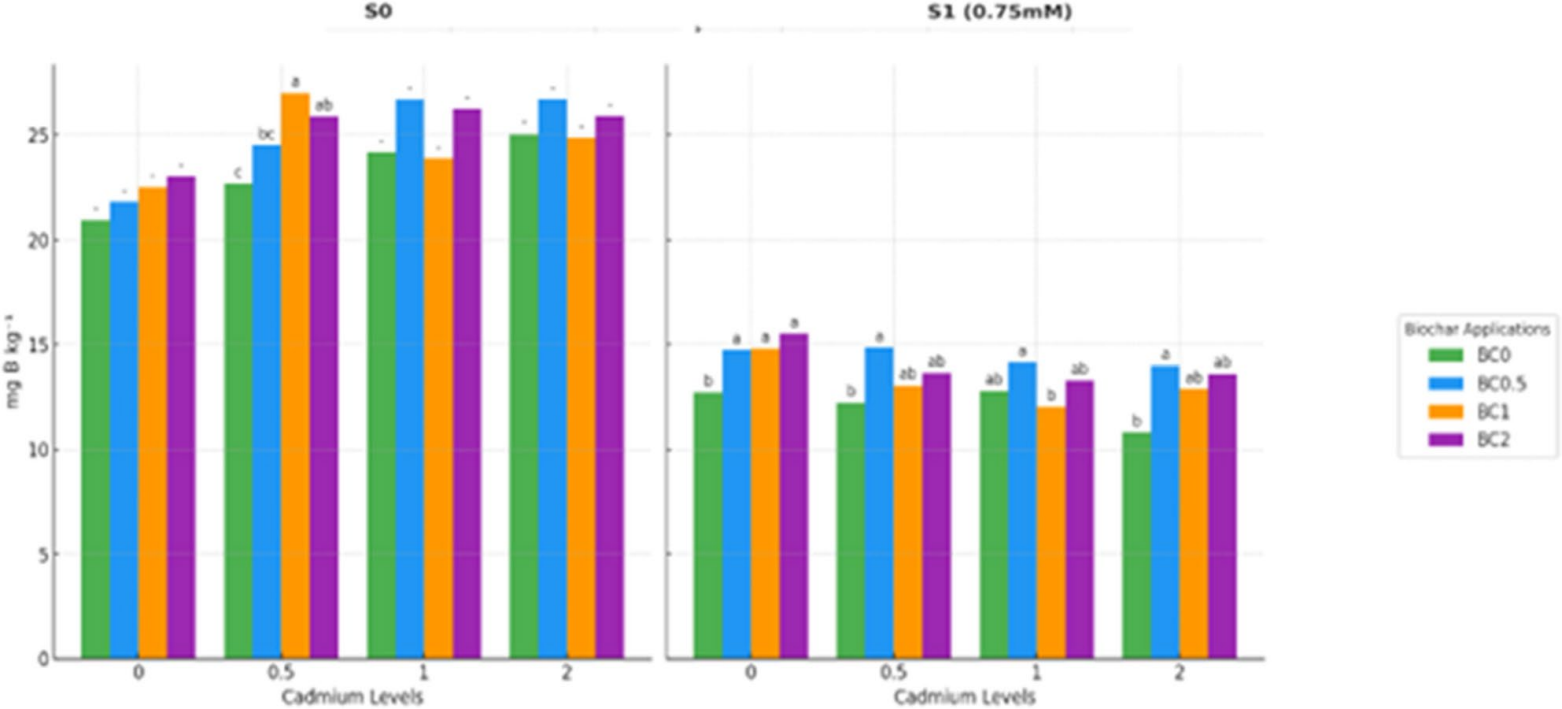
Boron concentration in plants under salt and cadmium stress: effect of biochar applications. Data followed by different letters were significantly different according to Duncan’s Multiple Range Test (*p* < 0.05; ns: *p* > 0.05)

Overall, these results demonstrate that boron uptake is strongly influenced by the interaction of biochar, Cd, and salinity. Biochar consistently improved B accumulation under both non-saline and saline conditions, with the magnitude of its effect depending on Cd concentration and the presence of salt stress. The data highlight the potential of biochar to counteract salinity-induced reductions in boron nutrition, particularly under moderate Cd exposure (Fig. 1).

**Fig. 2:**
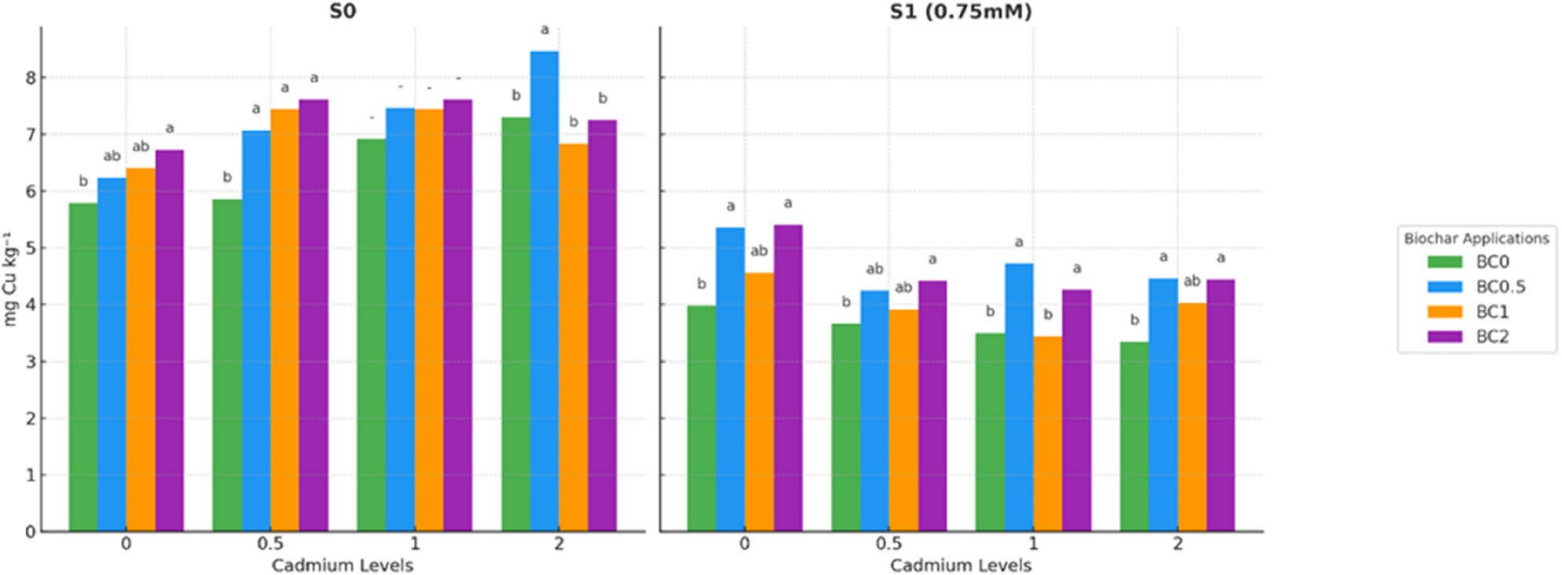
Copper concentration in plants under salt and cadmium stress with biochar applications. Data followed by a different letter were significantly different according to Duncan’s Multiple Range Test.(*p* < 0.05; ns: *p* > 0.05)

Significant differences were observed among the treatments (*p* < 0.05), demonstrating that the combined effects of biochar, Cd, and salt stress influenced copper nutrition in lettuce (Fig. 2). In S0, Cu concentrations increased with biochar application across all Cd levels. The lowest Cu content was observed in the Cd0 × BC0 treatment, measuring 5.79 ppm. Application of 2% biochar raised Cu to 6.73 ppm in the same Cd0 group, representing a 16% increase. At higher Cd levels, biochar further enhanced Cu accumulation. In the Cd0.5 treatment, Cu increased from 5.86 ppm without biochar to 7.61 ppm with 2% biochar, a 30% increase. Similarly, in Cd1, Cu rose from 6.92 ppm to 7.59 ppm, and in Cd2, biochar application resulted in a maximum Cu concentration of 8.47 ppm in BC0.5 × Cd2, indicating a strong synergistic effect of moderate Cd and biochar on Cu uptake.

Under S1 conditions, Cu concentrations were generally lower than in non-saline treatments, reflecting the inhibitory effect of salt stress. For instance, in the Cd0×BC0 group, Cu dropped to 3.98 ppm, representing a 31% reduction compared to non-saline conditions. Nevertheless, biochar mitigated this decline. In the Cd0 group, 0.5% biochar increased Cu to 5.36 ppm, while 2% biochar further improved Cu accumulation to 5.41 ppm. Similar trends were observed in Cd0.5, Cd1, and Cd2 treatments, with biochar raising Cu concentrations by 15–25% relative to the corresponding non-biochar controls. Overall, the results demonstrate that Cu uptake is strongly influenced by the interaction of biochar, Cd, and salinity. Biochar consistently enhanced Cu concentrations under both non-saline and saline conditions, although the magnitude of the effect was greater under non-saline conditions and at moderate Cd levels. These findings underscore the potential of biochar to alleviate salt-induced reductions in copper nutrition while modulating micronutrient uptake under heavy metal stress. Overall, biochar, Cd, and salinity interacted significantly to influence Fe nutrition. Non-saline conditions allowed higher Fe accumulation, while salinity stress generally reduced Fe levels. Biochar’s effects varied with Cd concentration, indicating complex interactions among micronutrient availability, heavy metal stress, and soil amendments.

**Fig. 3:**
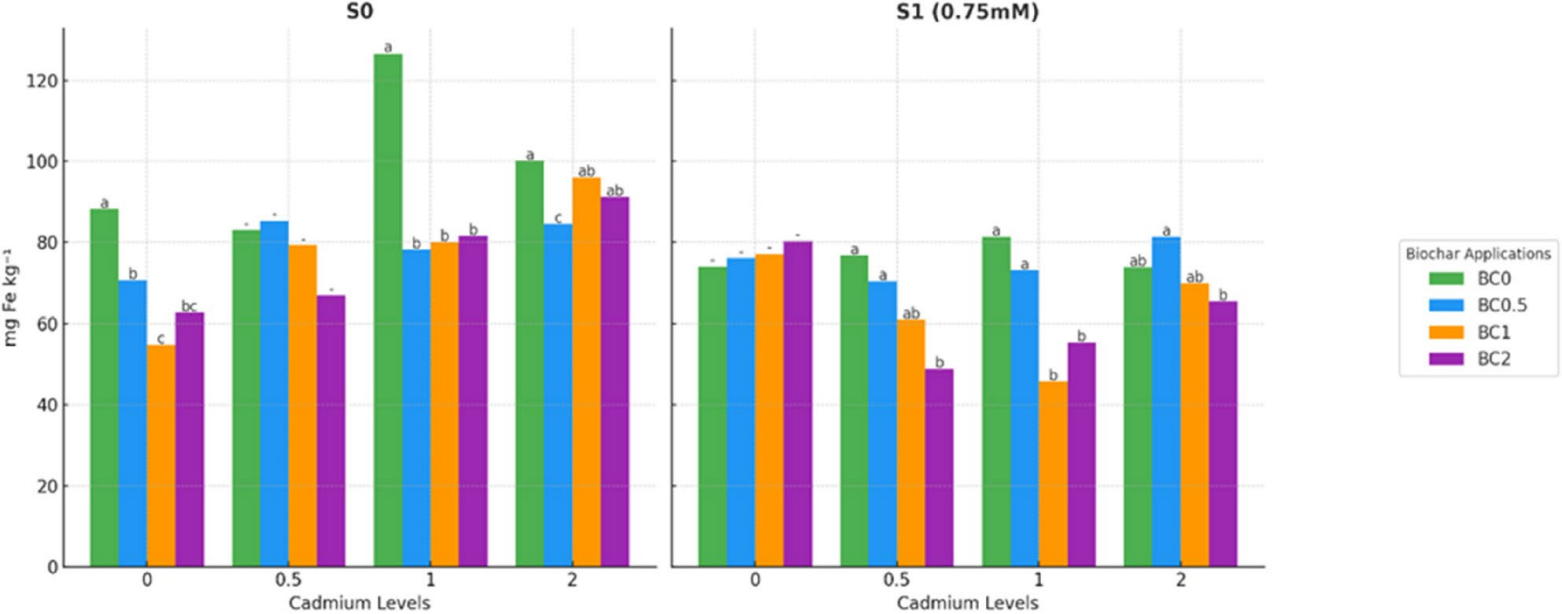
Iron concentration in plants under salt and cadmium stress: effect of biochar applications. Data followed by a different letter were significantly different according to Duncan’s Multiple Range Test (*p* < 0.05; ns: *p* > 0.05)

Statistically significant differences were observed among treatments (*p* < 0.05), demonstrating the interactive effects of biochar, Cd, and salt stress on iron nutrition in lettuce (Fig. 3). In S0 conditions, Fe concentrations varied depending on the combination of Cd and biochar. The lowest Fe levels were observed in BC1 × Cd0 (54.83 mg kg^− 1^), BC2 × Cd0.5 (66.96 mg kg^− 1^), BC0.5 × Cd1 (78.24 mg kg^− 1^), and BC0.5 × Cd2 (84.57 mg kg^− 1^). Conversely, the highest Fe concentrations were recorded in BC0 × Cd0 (88.23 mg kg^− 1^), BC0.5 × Cd0.5 (85.38 mg kg^− 1^), BC0 × Cd1 (126.61 mg kg^− 1^), and BC0 × Cd2 (100.12 mg kg^− 1^). These patterns indicate that biochar had a modulating effect, often reducing Fe concentrations at certain Cd levels, likely due to sorption interactions and competitive uptake with other cations.

Under S1 conditions, overall, Fe concentrations were lower, reflecting the suppressive effect of salt stress on Fe uptake. The lowest values were observed in BC0 × Cd0 (74.02 mg kg^− 1^), BC2 × Cd0.5 (48.80 mg kg^− 1^), BC1 × Cd1 (45.74 mg kg^− 1^), and BC2 × Cd2 (65.42 mg kg^− 1^), whereas the highest concentrations were recorded in BC2 × Cd0 (80.37 mg kg^− 1^), BC0 × Cd0.5 (76.70 mg kg^− 1^), BC0 × Cd1 (81.31 mg kg^− 1^), and BC0.5 × Cd2 (81.41 mg kg^− 1^). These findings indicate that under salt stress, higher biochar doses did not always lead to increased Fe accumulation, and the effect of biochar on Fe content was strongly influenced by the Cd concentration.

**Fig. 4:**
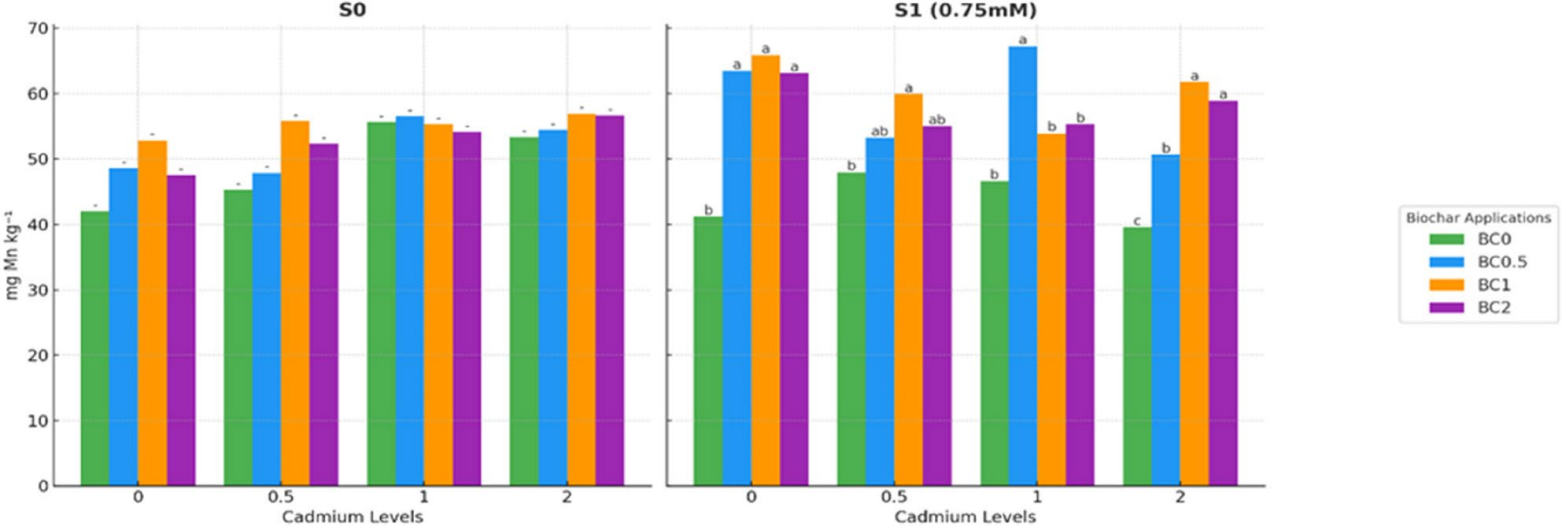
Manganese concentration in plants under salt and cadmium stress: effect of biochar applications. Data followed by a different letter were significantly different according to Duncan’s Multiple Range Test (*p* < 0.05; ns: *p* > 0.05)

In the S0 groups, Mn concentrations in lettuce plants did not differ significantly between treatments, suggesting that biochar and Cd had limited impact on Mn under optimal conditions. In contrast, under saline conditions (S1), Mn concentrations were significantly affected by the treatments (*p* < 0.05). The lowest Mn concentrations were found in the non-biochar treatments, specifically BC0×Cd0 (41.17 mg kg^− 1^), BC0×Cd0.5 (47.95 mg kg^− 1^), BC0×Cd1 (46.63 mg kg^− 1^), and BC0×Cd2 (39.56 mg kg^− 1^). In comparison, biochar application substantially increased Mn concentrations, with the highest values observed in BC1×Cd0 (65.90 mg kg^− 1^), BC1×Cd0.5 (59.93 mg kg^− 1^), BC0.5×Cd1 (67.25 mg kg^− 1^), and BC1×Cd2 (61.73 mg kg^− 1^). These differences were statistically significant, demonstrating that biochar effectively mitigated the adverse effects of salinity on Mn nutrition, and that the impact varied depending on the Cd level (Fig. 4).

**Fig. 5:**
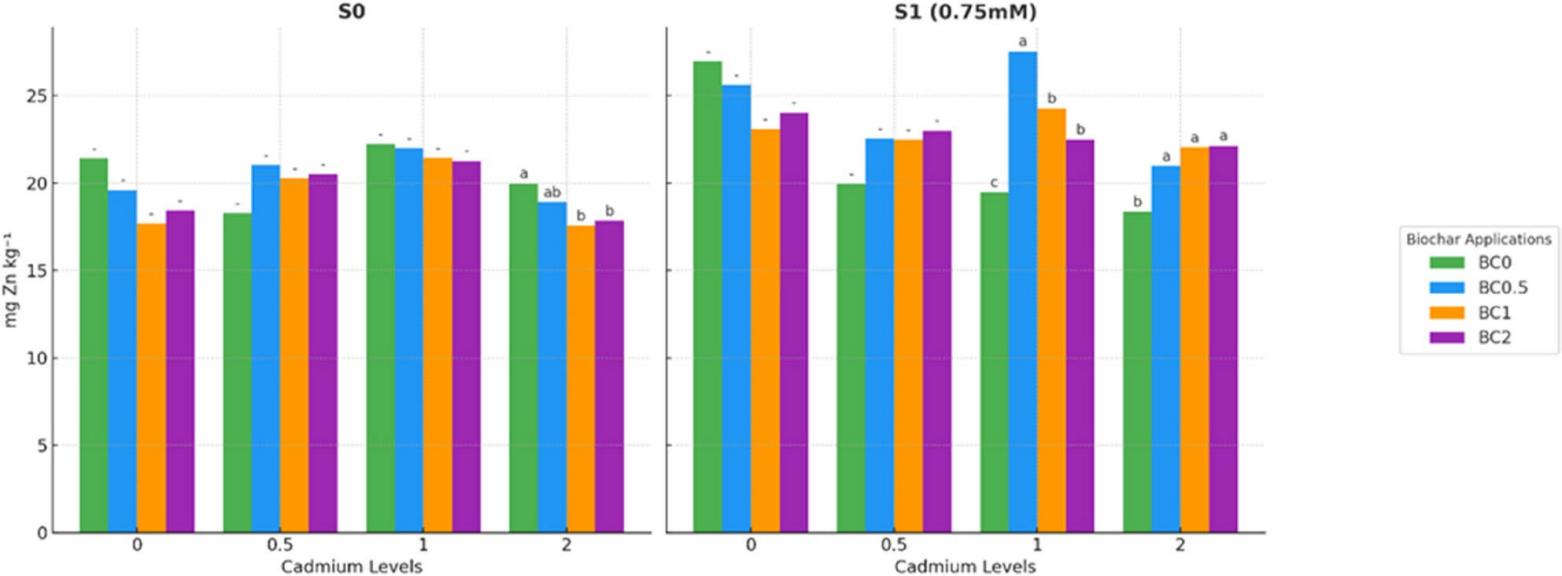
Zinc concentration in plants under salt and cadmium stress: effect of biochar applications. Data followed by a different letter were significantly different according to Duncan’s Multiple Range Test (*p* < 0.05; ns: *p* > 0.05)

Statistically significant differences were observed among the treatments (*p* < 0.05), demonstrating that biochar, Cd, and salt stress interacted to influence Zn concentrations in lettuce plants (Fig. 5). In the non-saline groups (S0), Zn concentrations were generally stable across most Cd and biochar treatments. For Cd0, Cd0.5, and Cd1, differences between biochar levels were minor and not statistically significant. However, at the highest Cd level (Cd2), Zn concentrations decreased with increasing biochar, with the lowest concentration recorded in BC1×Cd2 (17.54 mg kg^− 1^) and the highest in BC0×Cd2 (20.0 mg kg^− 1^), indicating that higher biochar dos slightly limited Zn uptake under elevated Cd stress.

Under S1 conditions, biochar application had a more pronounced effect on Zn concentrations. In the Cd1 treatments, BC0.5 increased Zn from 19.5 mg kg^− 1^ (BC0) to 27.5 mg kg^− 1^, representing a 41% increase, whereas in Cd2 treatments, BC2 enhanced Zn levels to 22.1 mg kg^− 1^ compared to 19.8 mg kg^− 1^ in BC1, indicating that biochar partially alleviated the combined inhibitory effects of Cd and salinity on Zn nutrition. Non-biochar treatments under salt stress consistently exhibited lower Zn concentrations, particularly in Cd0 (26.9 mg kg^− 1^) and Cd2 (18.3 mg kg^− 1^), highlighting the interactive influence of biochar, Cd, and salinity. These results suggest that moderate biochar application can improve Zn nutrition under combined Cd and salt stress, whereas excessive Cd or inappropriate biochar levels may limit this beneficial effect.

### Effects on macronutrients and Na⁺ accumulation

**Fig. 6:**
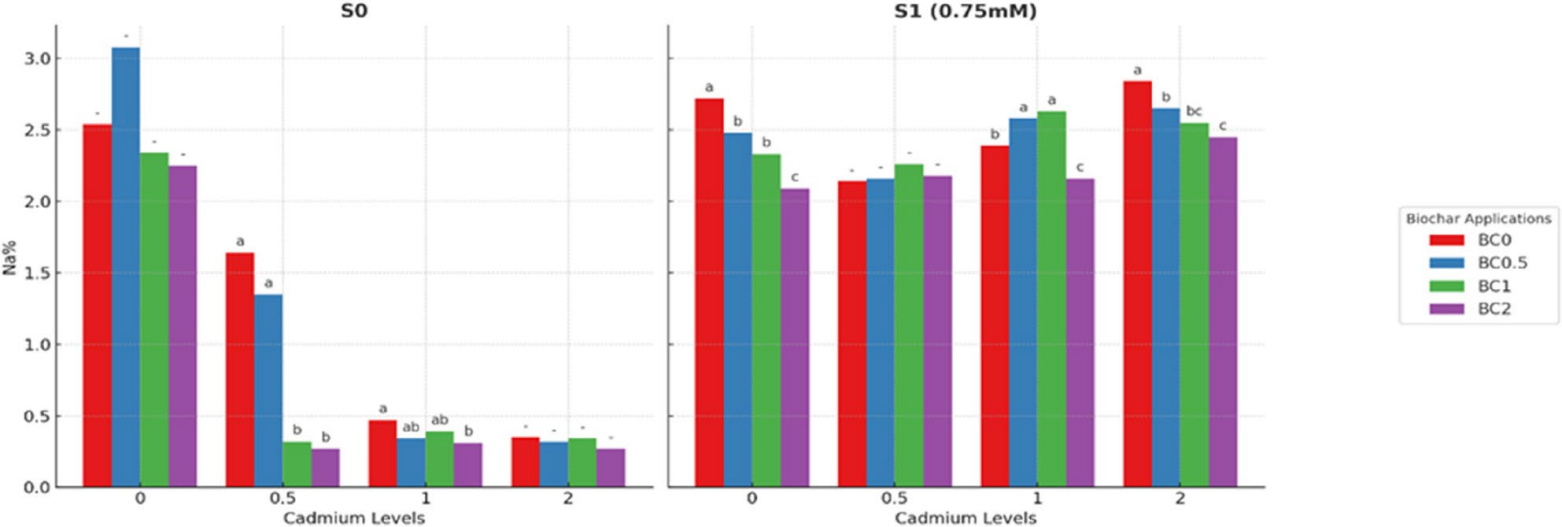
Na concentration in plants under salt and cadmium stress: effect of biochar applications. Data followed by a different letter were significantly different according to Duncan’s Multiple Range Test.(*p* < 0.05; ns: *p* > 0.05)

Na concentrations in lettuce were markedly influenced by biochar application, Cd levels, and salt stress (*p* < 0.05) (Fig. 6). In the BC0, Cd application led to a marked reduction in Na concentration. Under combined conditions, Na declined from 2.63 mg kg⁻¹ in the Cd0 control to 1.59 mg kg⁻¹ at Cd2, corresponding to a 40% decrease. When averaged across Cd levels, increasing biochar doses also reduced Na concentrations, with mean values decreasing from 1.89 mg kg⁻¹ at BC0 to 1.50 mg kg⁻¹ at BC2, representing a 21% reduction. Similarly, when averaged across biochar levels, Cd exerted a strong depressive effect on Na uptake, with concentrations dropping from 2.48 mg kg⁻¹ at Cd0 to 1.47 mg kg⁻¹ at Cd2, equivalent to a 41% reduction. Under S0 conditions, the Na concentration was generally low, but the combined effects of biochar and Cd were clearly pronounced. In the control treatment without biochar (BC0xCd0), Na concentration was 2.54 mg kg^− 1^. Under S0 conditions, Na concentrations were markedly reduced by the combined effects of biochar and Cd. In the control (BC0×Cd0), Na reached 2.54 mg kg^− 1^, but in BC2×Cd0.5 it declined to 0.31 mg kg⁻¹, corresponding to an 88% reduction. Comparable decreases were also observed in BC2xCd1 (0.34 mg kg⁻¹; 87% reduction) and BC2xCd2 (0.32 mg kg⁻¹; 87% reduction), indicating that biochar at the highest dose (2%) strongly suppressed Na accumulation in the presence of Cd. Under S1 conditions, Na concentrations were generally higher, yet biochar again exerted a mitigating effect. In the control treatment (BC0×Cd2), Na reached 2.84 mg kg^− 1^, whereas in BC2×Cd2 it decreased to 2.44 mg kg^− 1^, representing a 14% reduction. At Cd1 levels combined, Na concentrations in biochar-treated pots were 7–15% lower than in the BC0 counterparts. These findings suggest that, under non-saline conditions, the marked decline in Na concentration arises from the combined effects of Cd–Na competition during uptake and the sorptive capacity of biochar, which immobilizes both Cd and Na in the rhizosphere. This dual mechanism substantially limits Na accumulation in plant tissues. Under saline conditions (75 mM NaCl), Na concentrations increased as expected due to the external salt supply. However, biochar continued to mitigate Na accumulation by restricting its bioavailability and moderating the competitive interactions between Cd and Na, with BC2 treatments consistently showing lower Na levels than the respective controls.

**Fig. 7:**
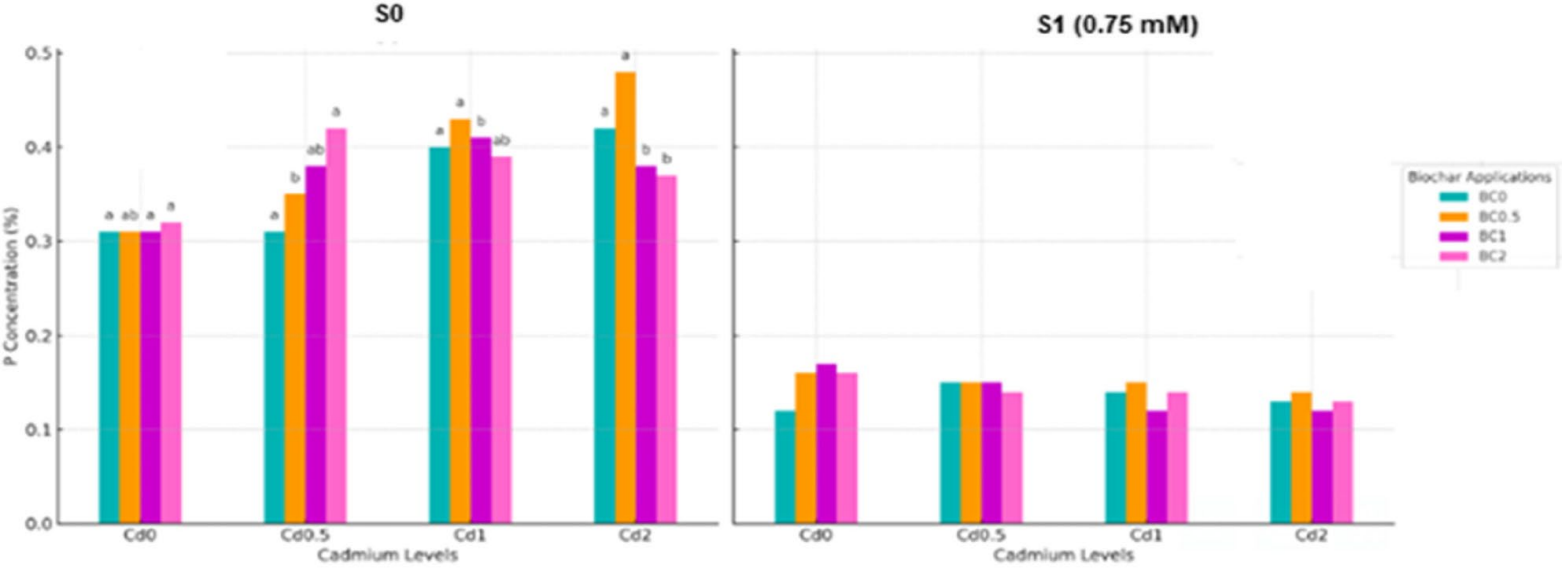
P Concentration in plants under salt and cadmium stress: effect of biochar applications. Data followed by a different letter were significantly different according to Duncan’s Multiple Range Test.(*p* < 0.05; ns: *p* > 0.05)

Phosphorus concentrations in lettuce were significantly influenced by the interaction of biochar, Cd, and salt stress (*p* < 0.05) (Fig. 7). In S0 conditions, the lowest P concentrations were observed in the control treatment without biochar (BC0×Cd0), measuring 0.31 mg kg^− 1^. With increasing Cd levels, P concentration generally rose, reaching 0.43–0.48 mg kg^− 1^ in BC2× Cd2, which corresponds to an approximate 38–55% increase compared with the Cd0 control. This increase was more pronounced in treatments with higher biochar doses, indicating that biochar enhanced P availability and uptake under non-saline conditions. Moderate Cd levels (Cd1) combined with biochar doses of 0.5–1% resulted in P concentrations of 0.41–0.43 mg kg^− 1^, statistically higher than the BC0xCd1 treatment.

Under S1 conditions, P concentrations were substantially lower compared with non-saline treatments. The lowest P levels were recorded in BC0×Cd0 (0.12 mg kg^− 1^) and remained nearly constant across biochar and Cd treatments, indicating that salt stress strongly limited P uptake. Even with biochar applications, P levels only reached 0.16 mg kg^− 1^ at maximum, reflecting a modest improvement compared with the control. Overall, P concentrations in lettuce were higher under non-saline conditions and were further increased by the combined application of biochar and moderate Cd doses. In contrast, under saline conditions, Cd and biochar had minimal effects, as high Na levels appeared to suppress P uptake. These findings demonstrate that biochar can improve P nutrition in lettuce, particularly in the absence of salt stress, while salinity imposes a strong limiting effect.

In both S0 and S1 conditions, Ca concentrations in lettuce were largely unaffected by BC or Cd treatments, with no statistically significant differences observed (SI.2). Within S0 groups, the lowest Ca levels were generally recorded in BC2 treatments, particularly in BC2×Cd0 (1.24 ± 0.19%), BC2×Cd0.5 (1.16 ± 0.12%), BC2×Cd1 (1.16 ± 0.12%), and BC2×Cd2 (1.18 ± 0.12%). Conversely, the highest Ca concentrations occurred in BC0.5 applications, including BC0.5×Cd0 (1.71 ± 0.00%), BC0.5×Cd0.5 (1.56 ± 0.22%), BC0.5×Cd1 (1.30 ± 0.04%), and BC0.5×Cd2 (1.34 ± 0.01%). Under S1 conditions, Ca concentrations remained relatively stable, with the highest levels observed in BC0×Cd0 (1.93 ± 0.20%), BC0.5×Cd0.5 (1.53 ± 0.26%), BC0.5×Cd1 (1.62 ± 0.14%), and BC1×Cd2 (1.68 ± 0.10%).

K concentrations exhibited greater variability in response to BC and Cd treatments. In S0 groups, K levels did not differ significantly among BC treatments for Cd0 and Cd0.5 applications; however, in Cd1 and Cd2 treatments, significant differences were observed (*p* < 0.05). The lowest K concentrations were recorded in BC0×Cd1 (6.11 ± 0.12%) and BC0xCd2 (6.27 ± 0.15%), while the highest were found in BC2×Cd1 (7.17 ± 0.02%) and BC1×Cd2 (6.80 ± 0.18%). Salinity substantially reduced K uptake, with the lowest concentrations in BC0 treatments (Cd0: 3.19 ± 0.45%, Cd0.5: 2.86 ± 0.21%, Cd1: 3.07 ± 0.12%, Cd2: 2.48 ± 0.15%), whereas BC2 applications mitigated this decline, yielding the highest K levels (Cd0: 3.95 ± 0.14%, Cd0.5: 3.64 ± 0.25%, Cd1: 3.46 ± 0.08%, Cd2: 3.46 ± 0.16%). These results indicate that BC application can partially counteract the negative effects of salinity on K nutrition in lettuce.

Mg concentrations were largely unaffected by BC in non-saline conditions. In S1 groups, however, Mg content differed significantly between Cd0 and Cd2 treatments, while no significant differences were observed for Cd0.5 and Cd1 (*p* < 0.05). Within these significant comparisons, the lowest Mg concentration was found in BC0×Cd0 (0.30 ± 0.03%), whereas the highest was recorded in BC0.5×Cd0 (0.34 ± 0.02%). Similarly, in Cd2 treatments, Mg concentrations ranged from 0.28 ± 0.02% (BC0) to 0.34 ± 0.02% (BC0.5), indicating that moderate biochar applications can help maintain Mg levels under saline stress.

### Principal component analysis of Biochar effects on cadmium and salinity stress

**Fig. 8:**
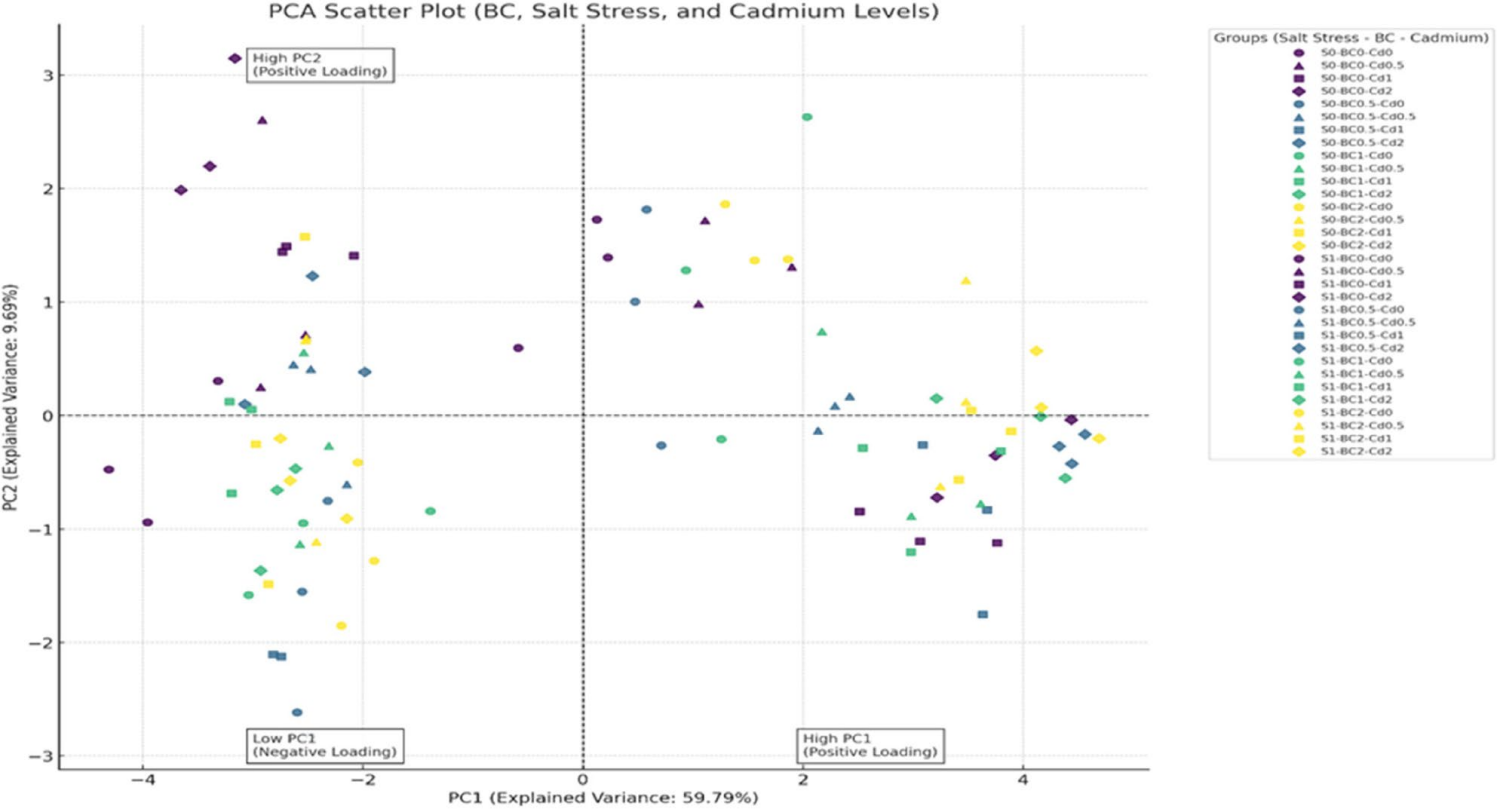
Principal Component Analysis (PCA) of plant nutrient dynamics under salt and cadmium stress with biochar applications

To explore the multivariate responses of lettuce to BC, Cd, and salinity (S) stress, a Principal Component Analysis (PCA) was conducted based on nutrient concentrations (P, K, Fe, Cu, Mn, B, and Cd). The first two principal components, PC1 and PC2, explained 59.79% and 9.69% of the total variance, respectively (Fig. 8). PC1 captured the main gradient of variation in nutrient concentrations across treatments, while PC2 reflected secondary variation, mainly associated with salinity stress. The large contribution of PC1 emphasizes the combined influence of biochar and cadmium on nutrient distribution, whereas PC2 indicates the additional effect of salinity.

The control group (S0–Cd0–BC0) was positioned in the negative regions of both PC1 and PC2, corresponding to low nutrient concentrations and the absence of stress. Treatments receiving higher biochar doses (BC1 and BC2) were located in the positive PC1 region, reflecting improved nutrient concentrations and a more balanced nutrient profile. In contrast, treatments with little or no biochar (BC0 and BC0.5) clustered in the negative PC1 region, indicating limited improvement in nutrient concentrations. Along with the PC2 axis, salinity-stressed treatments (S1), especially those combined with high cadmium concentrations (Cd2), shifted toward positive PC2 values, showing that salinity was the primary factor affecting nutrient concentrations. S0 treatments remained in the lower PC2 region, displaying relatively stable nutrient profiles. High-dose biochar (BC2) consistently shifted treatments toward the positive PC1 region and partially countered the negative effects of combined Cd and salinity stress. For instance, the S1–Cd2–BC2 treatment was clearly separated from S1–Cd2–BC0, indicating that biochar reduced sodium accumulation and enhanced concentrations of potassium and micronutrients under stress conditions. The PCA results reinforce the univariate findings by demonstrating that biochar, particularly at higher application rates, improves nutrient status and alleviates the impacts of Cd and salinity. Salinity emerges as a significant factor influencing nutrient variability along the second principal component (PC2). To gain further insight into how individual nutrients contribute to the principal components, the loading scores for PC1 and PC2 were examined. PC1 was predominantly driven by variables associated with overall nutrient enhancement resulting from biochar application, exhibiting strong positive loadings for P, K, Mn, Cu, and B, alongside a strong negative loading for Cd. This pattern indicates that increased nutrient concentrations are inversely associated with Cd accumulation. In contrast, PC2 was primarily shaped by salinity-related ionic imbalance, with Na showing a strong positive loading, while nutrients sensitive to salinity stress such as Zn and Fecontributed negatively. These loading patterns confirm that PC1 represents the gradient of nutrient enhancement and Cd reduction facilitated by biochar, whereas PC2 reflects the ionic stress imposed by salinity. Incorporating these loading relationships provides a clearer understanding of the treatment separation in the PCA biplot and supports the interpretation that biochar application shifts the nutrient profile toward improved plant nutrition, even under combined Cd and salinity stress.

## Discussion

This study investigated the influence of biochar amendments on lettuce (*Lactuca sativa* L.) subjected to varying Cd concentrations and salinity stress. The findings clearly demonstrate that biochar plays a multifaceted role in improving nutrient concentrations, mitigating Cd toxicity, and moderating salt-induced ionic imbalances. Similar multifunctional effects, simultaneously improving nutrient uptake, attenuating metal accumulation, and alleviating salinity-induced stress, have been reported for other organic amendments, such as silicon-enriched biochar and press mud (PM), in maize and wheat grown on salt-affected, metal-contaminated soils [56–58].

Under non-saline conditions, biochar substantially reduced Cd concentrations in lettuce shoots, with the most pronounced effect observed at the 2% application rate (BC2). In the Cd1 treatment, shoot Cd decreased by up to 93% relative to the control, confirming that biochar effectively immobilizes Cd in the soil through its large surface area and negatively charged functional groups [59]. In S1 conditions, however, elevated Na⁺ concentrations partly hindered Cd immobilization due to competition for adsorption sites, resulting in higher Cd levels in plant tissues compared to S0. These results emphasize that salinity substantially alters heavy metal behavior in soils, leading to a noticeable reduction in the capacity of biochar to immobilize Cd under saline conditions. Comparable patterns have been observed with Pressmud applied to Cd- and Pb-contaminated soils, where Pressmud significantly decreased Cd and Pb accumulation in edible grains and increased yield, indicating that different organic amendments can effectively lower the bioavailability of toxic metals while supporting crop performance [58]. Interestingly, in the non-amended treatments (BC0), an increase in Cd dosage did not lead to a decline in lettuce biomass. In fact, in some instances, it was associated with a slight increase in shoot fresh weight at the highest Cd level. This observation aligns with a hormetic response, where low to moderate Cd exposure prompts adaptive physiological adjustments that temporarily enhance growth and photosynthetic performance rather than causing immediate suppression. A similar case of Cd-induced hormesis has been documented in peppermint, where low Cd concentrations elevated biomass and photosynthetic pigments [60], as well as in a study, where sub-toxic Cd levels fostered growth and boosted antioxidant enzyme activities [61]. Therefore, the biomass response observed in BC0 likely indicates a phase of low to moderate Cd stimulation, which is compounded by the stronger mitigation effects provided by biochar at higher application rates.

Sodium dynamics further underscore biochar’s role in maintaining ionic balance. In non-saline soils, Na concentrations in lettuce declined sharply with BC addition. For instance, Na levels dropped from 2.54 mg kg⁻¹ in the BC0–Cd0 control to just 0.32 mg kg⁻¹ in the BC2–Cd2 treatment 87% reduction. The most striking decline occurred in BC2–Cd0.5, where Na concentrations fell by nearly 88%. These reductions cannot be explained by Cd competition alone; rather, they reflect a dual mechanism involving both Cd–Na competition during root uptake and the sorptive capacity of biochar, which immobilizes Na alongside Cd in the rhizosphere. Under saline conditions, Na concentrations increased as expected, but biochar consistently moderated this accumulation. In BC2–Cd2, Na was reduced to 2.44 mg kg⁻¹ compared with 2.84 mg kg⁻¹ in the corresponding BC0 treatment, indicating that biochar retained its protective role even under salt stress. These findings align with literature demonstrating biochar’s capacity to mitigate abiotic stresses by altering ion dynamics. Biochar’s high cation exchange capacity (CEC) and surface area enable strong adsorption of Na⁺, reducing its bioavailability and plant uptake under salinity [62, 63]. A comparable decrease in Na⁺ accumulation, along with enhancements in the uptake of K, P, and N, has been observed in maize when biochar is used in conjunction with silicon under saline irrigation conditions. The combined Si–biochar treatment significantly reduced Na⁺ levels and improved nutrient status, leaf area, and biomass in comparison to individual applications [57].This effect was particularly pronounced in non-saline treatments, where biochar appeared to enhance Cd-induced suppression of Na, potentially through competitive ion binding or increased root exclusion. Such interactions are consistent with [64], who reported that Cd²⁺ and Na⁺, as cations, can compete for uptake sites, suggesting that biochar may further modulate this antagonism by influencing the availability and mobility of these ions in the rhizosphere. Studies on wheat under salinity similarly report biochar binding Na⁺, improving hydration and nutrient status while alleviating osmotic stress. Regarding Cd-Na interactions, Cd toxicity often disrupts membrane integrity and ion homeostasis, reducing non-essential ion uptake like Na in non-saline environments through decreased transpiration or altered channel activity. The current study data support this, with sharp Na declines under Cd in S0 conditions, consistent with reports of Cd interfering with plasma membrane permeability and nutrient leakage, indirectly limiting Na influx [65].

The observed improvements in the K⁺/Na⁺ ratio, together with increased K availability, suggest enhanced ionic homeostasis and are consistent with previous reports of biochar reducing Na uptake and improving salt tolerance [66–69]. The application of microbial products in wheat systems affected by Pb contamination and salt stress has been shown to significantly enhance the uptake of N, P, and K by approximately 2 to 2.6 times, while also reducing Pb accumulation in grains by about 59 to 62%. This indicates that organic amendments can effectively restore nutrient balance and minimize the transfer of heavy metals to edible tissues in the presence of combined salinity and heavy metal stress [58]. Additionally, the study found that hazelnut husk biochar had a similar effect, increasing potassium and selected micronutrient concentrations while alleviating Na⁺ accumulation. This suggests that it operates in a manner comparable to microbial product-based strategies, promoting ionic and nutritional homeostasis under stress conditions.

Micronutrient dynamics also shifted in response to BC and Cd interactions. Iron concentrations decreased with higher BC levels under non-saline conditions, likely due to Fe immobilization on biochar surfaces, but increased under higher Cd stress, reflecting complex Cd–Fe interactions. Copper concentrations, in contrast, increased significantly with BC application, particularly under salinity, and showed a positive correlation with Cd in non-saline treatments. Manganese and boron concentrations remained relatively stable under S0 but rose significantly under S1 with biochar application, suggesting that BC enhances the availability of these micronutrients under osmotic and ionic stress. The negative correlations between Cd and Mn/B in saline soils likely result from ionic competition and reduced transporter efficiency, whereas positive associations under non-saline Cd stress indicate activation of metal transport systems [70–74]. These patterns agree with findings from PM-amended systems, where improved macronutrient uptake under metal-contaminated conditions coincided with reduced Pb and Cd accumulation in grains, suggesting that organic amendments can selectively promote nutrient acquisition while restricting toxic metal transport [58].

Phosphorus concentrations were consistently higher in non-saline soils but declined sharply under S1, particularly in Cd2 treatments. This reduction reflects osmotic stress, ionic imbalance, and reduced photosynthetic activity and transpiration under salinity [75–78]. Biochar’s influence on P was complex: while it improved nutrient retention in some treatments, it also potentially reduced P availability through surface binding or precipitation with Ca²⁺, particularly under saline irrigation 61. Similar context-dependent behavior has been observed with poultry manure and other organic amendments, where enhanced phosphorus retention can lead to improved soil fertility. However, under specific combinations of salinity and metal loads, sorption and co-precipitation processes may also limit phosphorus availability [56]. This highlights the significance of understanding the impact of hazelnut husk biochar on phosphorus dynamics, considering both its chemical binding capacity and the processes of plant uptake in multi-stress environments.

The multivariate patterns revealed by PCA provide further support for these findings. High BC doses (BC1 and BC2) clustered in the positive PC1 region, associated with improved nutrient concentrations, whereas low or no biochar treatments remained in the negative PC1 range, reflecting limited benefits. Salinity-stressed treatments, especially at Cd2, shifted toward positive PC2 values, underscoring the dominant role of salinity in shaping nutrient dynamics. Importantly, BC2 consistently mitigated combined Cd and salinity stress, as shown by the distinct separation of S1–Cd2–BC2 from S1–Cd2–BC0, confirming biochar’s buffering capacity against multiple stressors.

Although the present study did not incorporate direct surface characterization of the biochar–soil interface (e.g., FTIR, SEM–EDS, or BET analysis), the observed reductions in Cd and Na uptake by lettuce are fully consistent with well-established mechanisms. These include cation exchange, electrostatic attraction to negatively charged aromatic surfaces, complexation by oxygen-containing functional groups, and physical pore sorption. Together, these processes restrict the bioavailability of both toxic ions and help maintain nutrient homeostasis under the combined stress of salinity and heavy metal contamination. Future studies that include detailed physicochemical characterization of the biochar are encouraged, as they could further clarify the relative contributions of these interacting mechanisms.

Taken together, these results demonstrate that biochar can enhance nutrient concentrations, reduce Cd bioavailability, and alleviate Na-driven ionic imbalances in lettuce. Its benefits are greatest under S0 conditions but remain significant under salinity stress, particularly at higher application rates. By modulating both macro- and micronutrient concentrations-including Na, K, Ca, Mg, Fe, Cu, Mn, B, and P-biochar emerge as a strategic soil amendment to strengthen crop resilience under challenging environmental conditions. Future studies should evaluate long-term soil and plant responses, including impacts on microbial activity, antioxidant defense systems, and nutrient transporter regulation, as well as extend these findings to diverse crop species and genotypes.

## Conclusion

Biochar application effectively improved nutrient concentrations and mitigated cadmium toxicity in lettuce, with the strongest effects observed at the 2% application rate (BC2). Under non-saline conditions, biochar markedly reduced Cd concentrations, enhanced K⁺/Na⁺ balance, and promoted uptake of micronutrients such as Cu and Mn. Salinity stress partially limited the Cd-binding capacity of biochar, yet high-dose applications still alleviated Na⁺ accumulation and supported nutrient homeostasis. Principal Component Analysis confirmed that biochar positively influenced overall nutrient dynamics and buffered the combined effects of cadmium and salinity stress. These findings indicate that biochar is a valuable soil amendment for improving plant resilience under heavy metal contamination and salinity, with its benefits being most pronounced at higher application rates.

## Data Availability

The datasets generated and/or analyzed during the current study are available from the corresponding author on reasonable request.
